# Semantic and Phonological Abilities Inform Efficacy of Transcranial Magnetic Stimulation on Sustained Aphasia Treatment Outcomes

**DOI:** 10.1162/nol_a_00160

**Published:** 2025-03-07

**Authors:** Haley C. Dresang, Denise Y. Harvey, Leslie Vnenchak, Shreya Parchure, Sam Cason, Peter Twigg, Olu Faseyitan, Lynn M. Maher, Roy H. Hamilton, H. Branch Coslett

**Affiliations:** Department of Neurology, University of Pennsylvania, Philadelphia, PA, USA; Moss Rehabilitation Research Institute, Philadelphia, PA, USA; Department of Communication Sciences and Disorders, University of Wisconsin-Madison, Madison, WI, USA; Waisman Center, Madison, WI, USA; Department of Communication Sciences and Disorders, University of Houston, Houston, TX, USA

**Keywords:** constraint-induced language therapy, lexical retrieval, naming, neurorehabilitation, non-invasive brain stimulation, stroke aphasia

## Abstract

A growing body of evidence has shown that repetitive transcranial magnetic stimulation (rTMS) can enhance word-retrieval abilities in chronic aphasia. However, there remains significant variability in the efficacy of combined rTMS and language treatments. This study investigated how semantic and phonological characteristics of baseline word-retrieval impairments may influence the efficacy of rTMS on long-term naming improvements following language treatment in individuals with chronic aphasia. Thirty participants with post-stroke aphasia underwent 10 sessions of 1 Hz rTMS to right pars triangularis followed by a modified constraint-induced language treatment (mCILT). Nineteen participants were randomly assigned to active rTMS and 11 participants were assigned to sham rTMS. All participants completed the Philadelphia Naming Test (PNT) at baseline and at 3 and 6 months post-treatment. We coded PNT errors and fit data to the semantic-phonological (or SP) computational model (Foygel & Dell, 2000) to derive semantic and phonological parameter weights. We ran linear regressions for the proportional improvement in naming, with fixed effects for interactions between rTMS, time, and baseline parameter weights. While there was no immediate effect of rTMS post-treatment, rTMS combined with mCILT improved long-term naming more than language therapy alone. Furthermore, greater baseline semantic and phonological characteristics of word-retrieval abilities were each associated with increased rTMS-induced gains in proportional naming improvements. These patterns were maintained at both 3 and 6 months post-treatment. This study is among the first in a larger sample to demonstrate that individual differences in lexical retrieval contribute to variability in sustained rTMS and aphasia treatment outcomes.

## INTRODUCTION

Aphasia is the acquired loss of language abilities. The disorder affects approximately one in three stroke survivors and is among the most devastating long-term consequences of stroke (Berthier, 2005; Solomon et al., 1994). A growing body of evidence has shown that transcranial magnetic stimulation (TMS) can enhance naming abilities in chronic stages of post-stroke aphasia (see Gholami et al., 2022, for recent systematic review). TMS is a focal form of noninvasive brain stimulation in which an electromagnetic field penetrates the scalp and skull and induces a small electrical current in the brain that generates action potentials in neurons underlying the stimulation site. Repetitive TMS (rTMS) is a specific form of TMS that is administered as a series of electromagnetic pulses to modulate cortical excitability. When applied at low frequencies (<5 Hz), rTMS generally results in temporary cortical inhibition. Meta-analyses have reported significant positive effects of low-frequency rTMS on language function in post-stroke aphasia, including during chronic recovery stages (>6 months post-stroke; Hong et al., 2021; Li et al., 2015; Otal et al., 2015; Ren et al., 2014). Furthermore, low-frequency rTMS applied to contralesional cortex has shown promising effects as an adjuvant to language therapy (e.g., Harvey & Hamilton, 2022, for discussion).

Speech-language therapy is the clinical gold-standard treatment for aphasia. However, there are many forms of language therapies, and the efficacy of each therapy type is highly variable across individuals. In a large meta-analysis of randomized controlled trials across more than 3,000 subjects, Brady et al. (2016) reported that speech-language therapy improved functional outcomes compared to no intervention. However, the overall effect size was small (standardized mean difference of 0.28). These modest effect sizes highlight the need for adjuvant treatment approaches such as incorporating rTMS for aphasia rehabilitation.

Likewise, there is also significant variability in the efficacy of rTMS for the treatment of aphasia, and it remains unclear which individuals are good candidates for rTMS treatment approaches. One significant source of variability is lesion-related information, including overall lesion size and specific location of neural damage (Martin et al., 2009). In addition, person-specific variability in genetic and neurophysiological factors of neuroplasticity have been shown to contribute to variability in rTMS response (Dresang et al., 2022; Harvey et al., 2021; Parchure et al., 2022; Shah-Basak et al., 2020). Another important, yet understudied factor that may contribute to rTMS treatment efficacy is the psycholinguistic loci of aphasia language impairments.

Models of lexical word retrieval posit that naming deficits occur at two major stages: the translation of meaning to word form (i.e., semantics) and the translation of the word to its sound form (i.e., phonology; Dell et al., 2007). Harvey et al. (2019) reported preliminary evidence that rTMS delivered to the right pars triangularis (rPTr) in the inferior frontal gyrus enhances naming by facilitating phonological access during word retrieval. Specifically, in a sample of 10 participants with chronic aphasia, Harvey and colleagues found that low-frequency rTMS of rPTr reduced phonological but not semantic naming errors. One potential hypothesized mechanism for this is that inhibiting neuronal activity at rPTr may allow for increased activity of the left PTr (e.g., via reduced callosal inhibition). This may in turn strengthen the dorsal route for phonological access (Hula et al., 2020; Saur et al., 2008), resulting in reduced phonological naming errors. In particular, rPTr inhibition has been associated with a posterior shift in recruiting the right pars opercularis (rPOp), a neighboring region within Broca’s area homologue (Crosson et al., 2009). Unlike the rPTr, the rPOp is believed to be functionally homologous to its left hemisphere counterpart (Turkeltaub et al., 2011), playing a specific role in phonological processing (Gough et al., 2005; Halai et al., 2017). Thus, rPTr inhibition may enhance naming performance by facilitating the recruitment of the rPOp, thereby improving phonological access.

However, there is also evidence supporting the hypothesis that rTMS to rPTr supports naming by strengthening semantic access. In particular, Medina and colleagues (2012) found that rTMS of rPTr resulted in significant increases to lexical retrieval in discourse production, including increased number of unique nouns, total verbs, mean sentence length, and both open- and closed-class words per sentence (Medina et al., 2012). Although they did not examine phonological access, they found no differences in measures of grammatical complexity or sentence construction. These results suggest that rTMS of rPTr can improve lexical-semantic access, rather than selectively improving phonological access or syntactic abilities. By this account, inhibition of rPTr is hypothesized to reduce maladaptive overactivity of rPTr specific to semantic processing. This is broadly consistent with evidence that rPTr is not functionally homologous to left PTr semantic processing in persons with aphasia (Turkeltaub et al., 2011) and increased rPTr activation may actually be associated with overt naming errors (Postman-Caucheteux et al., 2010).

Taking these results together, evidence suggests that one or more stages of word retrieval may be influenced by rTMS of rPTr. Improvements in both stages of word retrieval—semantic and phonological access—have been proposed as potential mechanisms by which rTMS of rPTr facilitates successful naming. It remains unclear how participant profiles of semantic and phonological deficits pre-treatment may inform the efficacy of rTMS to rPTr on aphasia treatment outcomes. The goal of this current research study was to provide a preliminary characterization of participants with aphasia most likely to respond to rTMS during aphasia treatment. Furthermore, unlike many previous studies (e.g., Harvey et al., 2019), who employed a single session of rTMS, we report data from a clinical trial in which rTMS was administered for 10 days over 2 weeks. This increased number of sessions is common in rTMS aphasia treatment studies, with the goal of promoting long-lasting changes in naming and global language performance (Bucur & Papagno, 2019; Shah-Basak et al., 2016).

**Figure 1.**
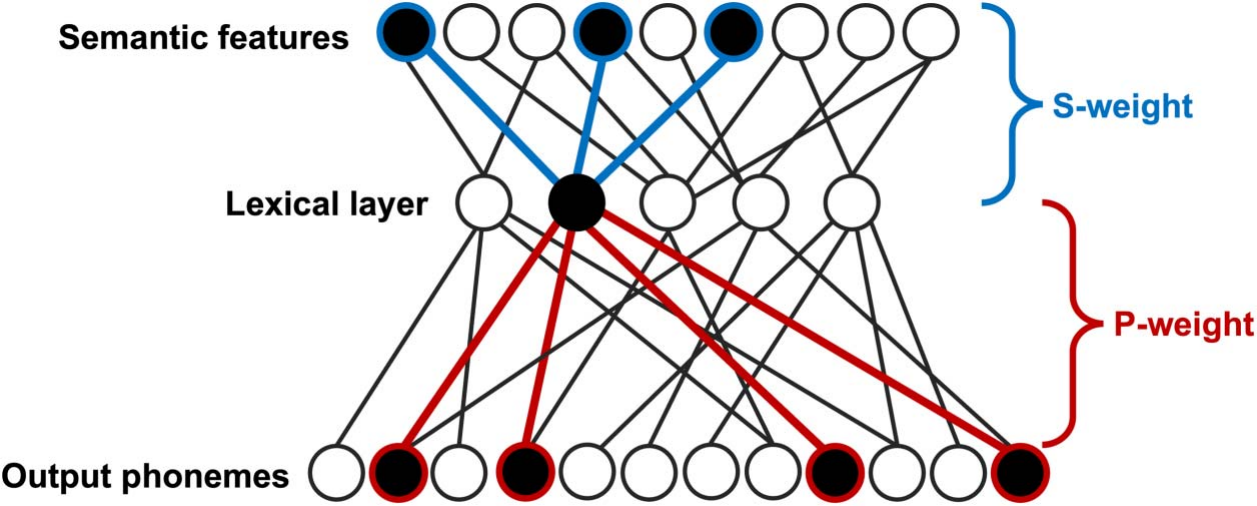
Interactive two-step model of lexical access. *S-weight* = semantic parameter; *P-weight* = phonologic parameter. This figure was adapted from computational psycholinguistic models of lexical access, such as Foygel and Dell (2000) and Walker and Hickok (2016).

The current study examined language deficits at the levels of semantic and phonological processing stages, as measured by the Philadelphia Naming Test (PNT; Roach et al., 1996). Computational psycholinguistic models relate an individual’s pattern of naming errors (i.e., paraphasias) to the integrity of semantic and phonological stages of lexical retrieval during picture naming (Foygel & Dell, 2000). This bidirectional two-step model is a localist connectionist network of three layers: semantic, lexical, and phonological. There are two lesionable parameters that determine the ability to map between layers of representations. First, *s-weight* reflects the strength of semantic ability, represented by connections between semantic features and lexical units. Second, *p-weight* reflects the strength of phonologic ability, represented by connections between lexical and phonological units. Figure 1 depicts the interactive two-step model of lexical access, including the network layers and the lesionable parameter weights that derive from them.

Following from Harvey et al. (2019), Medina et al. (2012), and previous language site-finding TMS studies (e.g., Garcia et al., 2013), the current study examined low-frequency, or inhibitory rTMS of the rPTr. Although the specific mechanism of action remains debated, prior work has consistently shown that inhibitory rTMS to rPTr enhances naming improvements in most adults with nonfluent aphasia (Barwood et al., 2011; Garcia et al., 2013; Hamilton et al., 2011; Naeser et al., 2005; Weiduschat et al., 2011).

Furthermore, there is evidence that benefits to picture naming not only are sustained but in fact increase over time, even in the absence of ongoing therapy (Barwood et al., 2013; Hamilton et al., 2010; Harvey et al., 2017; Naeser et al., 2005). However, neither immediate nor lasting (or enhanced) long-term benefits of rTMS can be guaranteed. Many prior findings of long-term benefit have been limited to small sample sizes with only nonfluent participants who were examined 1- or 2-months post-treatment, and it remains unclear how long any potential effects of rTMS may last. A prevailing hypothesis is that rTMS enhances use-dependent neuroplasticity, which can involve strengthening existing neural connections and forming new connections to regain function following stroke (Nudo, 2011). According to this hypothesis, neural circuits primed by rTMS may be further strengthened through continued use of the language system, thereby resulting in sustained language improvement. Even after completing rTMS or speech-language aphasia treatments, regular engagement of the language system may reinforce the neural pathways involved in naming, leading to progressive gains. It remains unclear under what circumstances, and through what mechanism, combined rTMS and aphasia treatments may support long-term improvements in language function. The current study therefore aimed to examine long-term naming outcomes that were measured months after rTMS and aphasia treatment was completed.

Use-dependent plasticity is also targeted in speech-language aphasia treatments such as constraint induced language therapy (CILT; Pulvermüller et al., 2001; various investigators have used alternate names for CILT, e.g., constraint induced aphasia therapy and interactive language aphasia therapy). Following the principles of constraint induced movement therapy (Kunkel et al., 1999; Miltner et al., 1999; Taub, 2004; Taub et al., 1999), CILT involves three key ingredients of use-dependent learning in stroke rehabilitation (Maher et al., 2006). First, massed practice of goal-based verbal language use occurs in an enriched environment. Second, alternative modes of communication such as gestures, drawing, and writing are not allowed to substitute for the spoken response. Third, use of spoken language production is required. CILT has shown significant improvements in communication quality, overall aphasia severity, naming, and narrative discourse in individuals with aphasia (Maher et al., 2006; Meinzer et al., 2007; Pulvermüller et al., 2001). Studies of CILT have reported that individual subjects can experience gains on multiple language behaviors, supporting its broad engagement of the language network, including both semantic and phonological stages of word retrieval (Martin et al., 2014; Meinzer et al., 2012). Here, we specifically aimed to target both semantic and phonologic processing to pair with rTMS based on the previously discussed evidence that rPTr stimulation may preferentially confer gains in these two pathways in a state-dependent manner. In this way, CILT is not expected to strongly bias results since both semantic and phonologic abilities are required during treatment.

For the current study, we combined CILT with rTMS, motivated by three primary considerations. First, prior evidence suggests that the combination of rTMS and behavioral therapies are better than rTMS alone, since rTMS can generate a brain state in which plasticity (and in theory, learning) is enhanced (Jannati et al., 2023). Specifically, following 20 minutes of 1 Hz rTMS, this state of enhanced plasticity persists for approximately 45 minutes (Avanzino et al., 2008). CILT was delivered immediately after rTMS to take advantage of these “state-dependent” effects of rTMS. This is critical because many of the previous studies examining effects of rPTr stimulation on language (e.g., Harvey et al., 2019; Medina et al., 2012) did not involve a language treatment that would maximize state-dependent effects. Second, Martin and colleagues (2014) reported benefit from CILT combined with rTMS in two participants with aphasia, establishing evidence in support of this specific combination approach. Finally, providing CILT ensures that all participants (i.e., even those who receive sham rTMS) receive an evidence-based aphasia treatment that applies to real-world transactional language goals.

In particular, this study investigated how semantic and phonological characteristics (*s-weight* and *p-weight*) of baseline naming impairments inform the efficacy of rTMS on long-term naming improvements following CILT in individuals with chronic aphasia. Although significant rTMS-related gains in naming accuracy have been reported (Li et al., 2015; Otal et al., 2015; Ren et al., 2014; Shah-Basak et al., 2016), they are not guaranteed. A likely source of substantial individual variability may be the psycholinguistic processes that support these gains. In this study, we evaluated naming performance before treatment and at 3 and 6 months post-treatment. We assessed the efficacy of combining low-frequency rTMS of rPTr with a modified version of CILT (mCILT), which is believed to enhance state-dependent neuroplasticity in aphasia (Maher et al., 2006). This research examined two questions: (1) Does low-frequency rTMS to rPTr paired with mCILT improve long-term naming more than mCILT and sham rTMS; and (2) Do individual baseline semantic and phonological abilities influence the efficacy of this rTMS treatment approach on long-term naming outcomes?

## MATERIALS AND METHODS

**Figure 2.**
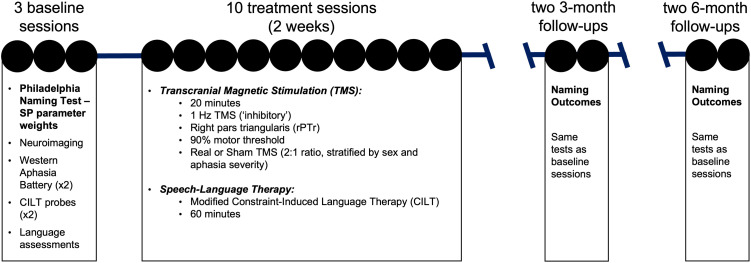
Study timeline. Circles indicate study session; bolded text indicates data examined in this study. SP = semantic-phonological.

Here we provide an overview of the experiment design (Figure 2), with detailed methodology presented below. All participants underwent baseline testing followed by ten 20-minute treatment sessions of inhibitory or sham rTMS to rPTr immediately followed by mCILT. This was a between-group clinical trial, whereby participants were randomly assigned in a 2:1 ratio to either active or sham rTMS conditions; randomization was stratified by aphasia severity. We conducted follow-up assessment sessions at 3 and 6 months post-treatment completion to evaluate long-term outcomes. Neuroimaging data were acquired at baseline and 6 months post-treatment; imaging was not analyzed for the purposes of this article. Furthermore, additional behavioral assessments from the larger study were not included here because they were not relevant to the current research question.

### Participants

Participants provided informed consent prior to participation, in accordance with the Institutional Review Board of Perelman School of Medicine at the University of Pennsylvania. Participants were 30 adults with aphasia following a single left-hemisphere ischemic stroke that occurred >6 months prior to participation. All participants were native English speakers with mild to very severe aphasia, operationalized as a Western Aphasia Battery—Revised Aphasia Quotient (WAB AQ; Kertesz, 2006; a global measure of language ability) between 20 and 85. No participants had a history of seizure within the past year, major psychiatric disorder, tinnitus, or drug or alcohol abuse. Participants agreed to not participate in any other therapies or interventions during their participation in the trial, including the 6-month follow-up period.

Of the 30 participants who completed the protocol, 19 were randomized to active rTMS and 11 were randomized to sham rTMS. See Table 1 for demographic data, grouped by rTMS condition. Two participants from the active rTMS group were excluded from analyses due to having minimal naming ability at baseline (Philadelphia Naming Test [PNT] accuracy <20%), leading to an insufficient number of meaningful errors to analyze. For example, one participant provided the same nonword response for the majority of trials; any phonological overlap with a target was thus random and not related to the specific word retrieval attempt. The final analyses therefore included a total of 28 participants.

**Table 1.** Participant demographic data

Study ID	TMS condition	Sex	Race	Ethnicity	Age at enrollment	Time post-stroke (yr)	Lesion volume (mm^3^)
TMSA-02	Active	Male	White	Non-Hispanic	55	8.23	100,465
TMSA-04*	Active	Male	White	Non-Hispanic	74	3.64	106,417
TMSA-06	Active	Male	White	Non-Hispanic	59	1.28	162,015
TMSA-15	Active	Female	White	Non-Hispanic	58	8.90	52,497
TMSA-20	Active	Female	Black/African American	Non-Hispanic	70	4.39	51,662
TMSA-24*	Active	Male	White	Non-Hispanic	58	1.63	123,916
TMSA-25	Active	Male	White	Non-Hispanic	63	15.32	181,110
TMSA-27	Active	Male	Asian	Non-Hispanic	56	6.54	89,648
TMSA-28	Active	Male	Black/African American	Non-Hispanic	57	1.08	18,551
TMSA-36	Active	Male	White	Non-Hispanic	65	1.89	44,647
TMSA-39	Active	Female	Other	Undisclosed	61	1.51	24,129
TMSA-40	Active	Male	White	Hispanic	66	1.14	74,465
TMSA-44	Active	Male	White	Non-Hispanic	68	2.53	79,430
TMSA-52	Active	Female	White	Non-Hispanic	60	0.53	67,118
TMSA-54	Active	Male	White	Non-Hispanic	36	4.52	230,789
TMSA-55	Active	Male	White	Non-Hispanic	70	3.51	106,909
TMSA-58	Active	Male	White	Non-Hispanic	74	1.41	17,794
TMSA-59	Active	Female	White	Non-Hispanic	52	2.05	54,629
TMSA-63	Active	Female	Black/African American	Non-Hispanic	36	15.30	249,644
Active TMS condition, summary statistics, mean (standard deviation)					60 (10.57)	4.5 (4.51)	96,623 (67,287)
TMSA-11	Sham	Male	White	Non-Hispanic	53	2.56	190,152
TMSA-13	Sham	Male	Black/African American	Non-Hispanic	61	7.97	26,046
TMSA-19	Sham	Male	White	Non-Hispanic	74	20.04	285,612
TMSA-26	Sham	Male	White	Non-Hispanic	77	10.97	109,036
TMSA-33	Sham	Male	White	Non-Hispanic	49	1.57	175,928
TMSA-35	Sham	Male	Bi-/Multi-Racial	Non-Hispanic	58	13.05	38,668
TMSA-38	Sham	Male	White	Non-Hispanic	69	0.82	152,235
TMSA-43	Sham	Male	Other	Undisclosed	72	1.71	23,455
TMSA-47	Sham	Female	Black/African American	Non-Hispanic	64	1.07	2,182
TMSA-48	Sham	Male	White	Non-Hispanic	63	1.11	202,486
TMSA-64	Sham	Male	White	Non-Hispanic	36	7.84	38,770
Sham TMS condition, summary statistics, mean (standard deviation)					61 (12.05)	6.25 (6.35)	113,143 (93,896)

* Participants were excluded from analysis due to having minimal naming ability at baseline (PNT < 20% accuracy), from which there were insufficient meaningful errors and too few items produced for an accurate word-retrieval sample selection.

PNT = Philadelphia Naming Text (Roach et al., 1996); TMS = transcranial magnetic stimulation.

### Language Assessments

The PNT (Roach et al., 1996) is a 175-item test of common object picture naming designed to measure retrieval of known object words (nouns). Computational models relate an individual’s PNT accuracy and error breakdown to the integrity of semantic and phonological stages of naming (SP model; Foygel & Dell, 2000; Walker & Hickok, 2016). The PNT thus produces a psycholinguistic diagnosis of word production impairments. As there was no overlap between target words in the PNT and words that were treated in therapy, changes in PNT accuracy may be viewed as a measure of generalization from treatment.

Participants completed the PNT at pre-treatment baseline and at 3 and 6 months post-treatment. We coded PNT errors following established guidelines (Schwartz et al., 2006), in which the first complete attempt at naming each picture was assigned one of six categories. Naming attempts were coded as correct, semantic errors, phonological (formal) errors, mixed errors (both semantically and phonologically related), unrelated real word errors, and nonword errors. Following the PNT scoring guidelines, we applied the lenient scoring procedure for participants with a motor speech disorder (e.g., dysarthria, apraxia of speech), such that individuals were allowed one sound omission, addition, or substitution per response when considering correctness. Two clinical speech-language pathologists, who were experienced with aphasia and motor speech disorders, determined whether motor speech deficits were present for each participant (Table 2). Each participant’s response counts were fit to the semantic-phonological computational model (Foygel & Dell, 2000; Walker & Hickok, 2016) to derive estimates of semantic and phonological abilities per subject (i.e., *s* and *p* parameters). We specifically used the *s* and *p* parameters from the Walker and Hickok (2016) web-based SLAM Model (semantic-lexical-auditory-motor model) application. Parameter values range from 0 to 0.04, with 0.04 considered normal function.

**Table 2.** Participant assessment data

Study ID	Baseline						3 months post-treatment		6 months post-treatment	
	Motor speech	WAB class	WAB AQ	PNT	*S* parameter	*P* parameter	PNT	Proportion improvement	PNT	Proportion improvement
TMSA-02	yes	Broca’s	76.4	0.87	0.0331	0.0232	0.93	0.4091	0.93	0.4545
TMSA-04*	yes	Broca’s	30.2	0.01	0.0331	0.0007	0.02	0.0115	0.02	0.0115
TMSA-06	yes	Conduction	84.3	0.91	0.0331	0.0331	0.93	0.2000	0.93	0.2000
TMSA-15	yes	Conduction	75.1	0.74	0.025	0.0238	n/a	n/a	0.87	0.5349
TMSA-20	yes	Conduction	68.7	0.78	0.0394	0.0182	0.77	−0.0256	0.75	−0.1282
TMSA-24*	yes	Broca’s	19.5	0	0.04	0.0001	0	0	0	0
TMSA-25	yes	Conduction	77.6	0.86	0.0325	0.0213	0.85	−0.0800	0.85	−0.0800
TMSA-27	yes	Broca’s	38	0.23	0.0157	0.0132	0.14	−0.1185	0.24	0.0148
TMSA-28	no	Conduction	66.4	0.39	0.0151	0.0163	0.40	0.0187	0.36	−0.0467
TMSA-36	yes	Conduction	79.7	0.86	0.0331	0.0219	0.86	0	0.91	0.3600
TMSA-39	yes	Transcortical motor	76	0.77	0.0394	0.0157	0.84	0.3000	0.88	0.4750
TMSA-40	no	Anomic	82.9	0.61	0.02	0.025	0.64	0.0870	0.62	0.0290
TMSA-44	yes	Anomic	78.3	0.91	0.0381	0.0282	0.86	−0.6667	0.89	−0.2667
TMSA-52	no	Anomic	81	0.89	0.04	0.0275	0.87	−0.2105	0.92	0.2632
TMSA-54	yes	Broca’s	62.1	0.85	0.035	0.0263	0.88	0.1923	0.93	0.5385
TMSA-55	yes	Transcortical sensory	51	0.51	0.0176	0.0225	n/a	n/a	0.31	−0.4070
TMSA-58	yes	Broca’s	61.1	0.47	0.0169	0.0194	0.39	−0.1522	0.52	0.1160
TMSA-59	yes	Anomic	82.3	0.95	0.0394	0.0294	0.93	−0.1513	0.96	0.1250
TMSA-63	yes	Conduction	72.5	0.78	0.0275	0.0213	0.89	0.4872	0.91	0.5897
Active TMS summary statistics, mean (standard deviation)			66.48 (18.88)	0.65 (0.3)	0.030 (0.009)	0.020 (0.009)	0.66 (0.3)	0.02 (0.27)	0.67 (0.3)	0.15 (0.29)
TMSA-11	yes	Broca’s	32.7	0.41	0.0213	0.0188	0.38	−0.0583	0.34	−0.1165
TMSA-13	no	Anomic	85.3	0.91	0.0388	0.0213	n/a	n/a	0.86	−0.5625
TMSA-19	yes	Broca’s	43.7	0.42	0.0151	0.025	0.43	0.0196	0.44	0.0392
TMSA-26	no	Wernicke’s	46.4	0.67	0.0213	0.02	0.71	0.1207	0.72	0.1552
TMSA-33	yes	Broca’s	47	0.31	0.0132	0.0331	0.39	0.1240	0.37	0.0909
TMSA-35	no	Anomic	83.8	0.95	0.0394	0.0294	0.93	−0.5000	0.93	−0.5000
TMSA-38	yes	Anomic	79.6	0.81	0.0394	0.0194	0.85	0.1818	0.81	−0.0303
TMSA-43	no	Anomic	74.7	0.25	0.0119	0.0257	0.30	0.0611	0.33	0.1069
TMSA-47	yes	Anomic	75.7	0.57	0.0188	0.0288	n/a	n/a	0.86	0.6667
TMSA-48	yes	Broca’s	65.4	0.56	0.0244	0.0269	0.57	0.0260	0.67	0.2468
TMSA-64	no	Anomic	83.4	0.86	0.0375	0.0238	0.90	0.2500	0.94	0.5833
Sham TMS summary statistics, mean (standard deviation)			65.25 (19.22)	0.61 (0.3)	0.026 (0.011)	0.025 (0.005)	0.61 (0.3)	0.02 (0.22)	0.66 (0.2)	0.06 (0.38)

*Note*: Motor speech is apraxia of speech or dysarthria, as judged by two independent speech-language pathologists.

* Participants were excluded from analysis due to having minimal naming ability at baseline (PNT < 20% accuracy), from which there were insufficient meaningful errors and too few items produced for an accurate word-retrieval sample selection.

WAB AQ = Western Aphasia Battery—Revised Aphasia Quotient (Kertesz, 2006).

The level of mCILT treatment achieved varied by each individual participant’s abilities (Table 3). To assess pre-treatment baseline and immediate post-treatment naming, we measured noun accuracy naming performance on the mCILT naming cards used during therapy (further details in the Modified Constraint Induced Language Therapy section, below). Blinded raters reviewed audio recordings to assess response accuracy for all trained agent nouns. This mCILT noun accuracy thus provides a measure of each participant’s change in naming performance, based on items directly treated during therapy. This analysis intends to demonstrate direct treatment effects by assessing changes immediately from pre- to post-treatment, whereas additional confounding environmental and social factors (Harvey et al., 2021) could be introduced between treatment and 3- or 6-month follow-up points.

**Table 3.**
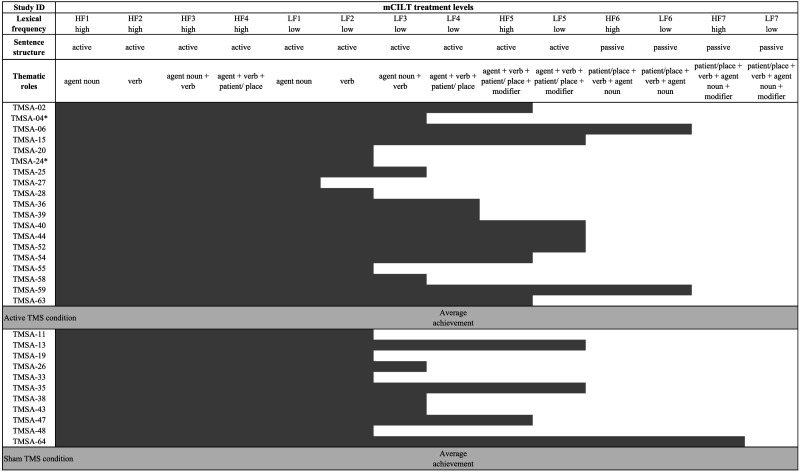
Participant modified Constraint Induced Language Therapy (mCILT) levels at start and end of treatment

*Note*: Average level of achievement for both active and sham rTMS randomization groups were level L4, low frequency agent + verb + patient/place.

HF = high-frequency agent noun; LF = low-frequency agent noun.

* Participants were excluded from analysis due to having minimal naming ability at baseline (PNT < 20% accuracy), from which there were insufficient meaningful errors and too few items produced for an accurate word-retrieval sample selection.

All participants also completed the WAB (Kertesz, 2006). The WAB provides a measure of overall aphasia severity by sampling multiple language functions that are used together to compute an AQ summary score between 0 and 100, with a clinical cutoff for aphasia <93.8. The WAB specifically evaluates linguistic content, fluency, auditory comprehension, repetition, and naming domains. WAB AQ was used to determine eligibility for the study and to characterize the overall aphasia severity of the sample.

### Neuroimaging

All participants underwent high-resolution brain magnetic resonance imaging (MRI) scans on a Siemens 3T Prisma before and 6 months after treatment. The MRI scan protocol included high-resolution T1-weighted scans (1 × 1 × 1 mm voxels), pseudo-CASL perfusion imaging, high-resolution diffusion imaging (HARDI), and fluid attenuated inversion recovery (FLAIR). Brain images were loaded into Brainsight software (Rogue Research, 2025) and were used to guide rTMS administration. To characterize the extent and location of lesions, we followed established lesion tracing procedures that are outlined in Schnur et al. (2009) and employed in numerous other studies (e.g., Schwartz et al., 2011, 2012).

### Repetitive Transcranial Magnetic Stimulation Treatment

In the treatment phase, there were ten rTMS sessions over 2 consecutive weeks. TMS was administered using a MagPro X100 with MagOption stimulator, equipped with an active cooling, 70 mm figure 8, Cool-B65 active/placebo coil manufactured by Magventure. Participants were seated in a comfortable chair with head support, and the coil was placed at a 45 degree angle tangential to the rPTr. For each session, 20 minutes (1200 pulses) of 1 Hz rTMS at 90% resting motor threshold (MT) were delivered to the right inferior pars triangularis. Each rTMS treatment session was immediately followed by the mCILT session. Due to substantial variability with respect to the anatomy of the inferior frontal gyrus (Keller et al., 2009), the site to be stimulated in each subject was identified by a neurologist (H.B.C.) with over 20 years of experience in prior studies of the effect of TMS on naming when delivered to the rPTr. Prior work exploring TMS to different sites within and around Broca’s area homologue reveals that the majority of people with aphasia show greatest naming improvement following 1 Hz rTMS to the inferior portion of the rPTr (e.g., Medina et al., 2012; Naeser et al., 2005). Based on these findings, a site in the inferior portion of the rPTr was selected as the target stimulation site on each subject’s structural MRI in native space. Following prior work, we used 1 Hz stimulation at 90% resting MT (Barwood et al., 2013; Hamilton et al., 2010; Martin et al., 2004; Medina et al., 2012; Naeser et al., 2010). MT was determined in the beginning of each session using standard procedures (Rossi et al., 2021; Rossini et al., 1999). The sham rTMS group was treated in the same manner as the experimental group except that MT was not determined for the sham group.

Participants were randomly assigned in a 2:1 ratio to either active or sham rTMS conditions. As it has been suggested that aphasia severity influences response to rTMS treatment for aphasia (e.g., Harvey et al., 2019), randomization was stratified by WAB AQ score to ensure an approximately equal allocation of rTMS treatments among participants with WAB AQ <45 and WAB AQ ≥45 (Kernan et al., 1999). Participants were also stratified by sex in a 1:1 ratio to either active or sham rTMS conditions to achieve balance across groups. Participants were not informed of their assignment to active or sham rTMS. Although individuals with experience with rTMS may distinguish real from sham rTMS, subjects with no prior rTMS experience have no basis for comparison and cannot distinguish real from sham rTMS. No participants in this study had prior experience with rTMS. Sham rTMS was administered with a sham rTMS coil that looks and sounds like the active coil but does not generate a magnetic field. The individuals administering rTMS were aware of group assignment, but all other individuals in contact with the subject or their data were blinded to group assignment until data analysis. In particular, the individuals delivering therapy and administering and scoring the language measures were not present for the administration of rTMS and were blinded with respect to subject assignment.

### Modified Constraint Induced Language Therapy

Immediately following each rTMS session, we administered mCILT (Pulvermüller et al., 2001), which invokes use-dependent language learning by requiring spoken output and restricting use of alternative communication forms, such as gestures, as substitutes for the spoken response. Therapy was administered by a certified speech-language pathologist or trained staff member under the supervision of the speech-language pathologist (L.V.), referred to henceforth as “therapy administrator.” Treatment fidelity was maintained by using a treatment protocol checklist during training until consistency was established. Recorded sessions were reviewed periodically using the treatment protocol checklist (see Table S1 in the Supporting Information, available at https://doi.org/10.1162/nol_a_00160), and any deviations from the protocol were identified and corrected for subsequent sessions.

mCILT stimuli comprised 72 cards, where 36 depicted agent nouns with high spoken lexical frequency and 36 depicted agent nouns with low spoken lexical frequency. All images illustrated an agent noun (a person) performing a semantically related action (verb) on an object (patient) or at a location (place). There was no overlap between the mCILT stimuli and the stimuli of the WAB or PNT.

Performance at baseline on the two 36-item sets was used to inform the selection of treated and untreated items. Each set was presented twice in the baseline sessions (see Figure 2), with participants scored on their ability to name the agent in the image and use the name in a sentence. Phonemic, semantic, unrelated, self-correction, and no response errors were transcribed and coded from audio transcripts. We then selected 24 high-frequency agent noun cards and 24 low-frequency agent noun cards to be used during treatment. The treatment items were individualized for each participant, where the assignment to treated and untreated sets were balanced for the consistency with which each participant accurately retrieved the agent noun across two baseline sessions, agent noun and verb spoken lexical frequency (as determined by the Corpus of Contemporary American English; Davies, 2008), and agent noun and verb name agreement (as determined by Amazon Mechanical Turk [MTurk], a crowdsourcing marketplace).

We used a dual card-matching task modeled after Maher and colleagues (2006). Typically, CILT is provided in small groups, but because we could not administer stimulation to more than one participant at a time, we modified the original protocol to deliver individual therapy with the therapy administrator serving as the communication partner. In addition, the task difficulty hierarchy was modified to include semantically associated nouns and verbs and sentences at higher levels of difficulty, with the goal of engaging all levels of the language system. As in the original CILT design, the participant interacts verbally with a conversation partner (here, the therapy administrator), in turn requesting a card that contains an image designed to elicit a sentence response and complying with the partner’s request. In this way, the treatment targets both production and comprehension. Furthermore, as treatment progressed, the verbal targets increased in length and linguistic complexity following the standard CILT protocol of scaffolding from simpler to more complex utterances. For example: “Do you have *the jester*?”, “Do you have *the jester is juggling*?”, “Do you have *the jester is juggling balls*?”, “Do you have *the jester is juggling green balls*?”, “Do you have *the green balls were juggled by the jester*?” The treatment difficulty scaled from producing high-frequency agents to phrases and sentences, followed by low-frequency versions of the same, to active sentences with modifiers, and when possible, passive sentence structures. See Table 3 for parameters of each mCILT level and the level that each participant achieved.

In each mCILT session, the participant and therapy administrator started with identical decks of eight treatment cards per “game.” Seated across a 30-cm barrier, each person was dealt four cards, arranged in an array in front of them. Choosing a card from their array, the participant would request its match by using a verbal description (e.g., “Do you have *the jester*?”). Following Stahl et al. (2018), nonverbal expressions of the target, though not prohibited, would not be accepted as a substitute for the verbal response. The therapy administrator would then take a turn by requesting a card from the subject. If the participant or therapy administrator did not receive a match to their card, they had to pick up a card from the deck, in similar fashion to the game Go-Fish. Once all eight cards in the set have been played, the game is repeated with the next set of cards.

The mCILT administration started at the same level for all participants (i.e., high-frequency agent nouns) and was subsequently scaled to individual ability based on criterion performance and individual rate of improvement. Once all sets of 24 cards achieved ≥75% accuracy twice, the participant was advanced and the procedure was repeated at the next level of difficulty (e.g., requesting agent nouns to requesting verbs, and so on). Participants were probed using a 10-item subset of treated items randomly chosen at the start of the 3rd, 6th, and 9th treatment day to monitor progress. Following the probes, treatment advanced to the next level of difficulty, even if the criterion benchmark was not achieved. This was done so that participants who struggled with one level would still get exposure to the stimuli at the higher levels of difficulty and encourage stimulus variety for each participant. Accuracy was determined by producing the target correctly without requiring a cue from the administrator.

The goal for each session was for each set of cards to be played twice. Due to the range of aphasia severity, some participants (e.g., those with more severe aphasia) required more time to get through all the treated items. Participants who achieved criterion on all the sets at one level during the session were advanced to the next level for the rest of the session. Each mCILT session therefore lasted 60–90 minutes to allow each participant their own pacing to achieve an equal amount of practice and stimulus exposure. The variability of session time is a potential limitation of the study, but the priority of the research was to equilibrate exposure to items across subjects. Although mCILT is often administered using a high intensity protocol (Meinzer et al., 2004), the optimal schedule of delivery is undetermined (Ciccone et al., 2016; Mozeiko et al., 2016). In this study, each participant received a minimum of 10 to a maximum of 15 hours of therapy.

### Statistical Analysis

Two participants from the active rTMS group were excluded from analysis due to baseline production of one or fewer correct items on the PNT, rendering their *s-* and *p-weights* meaningless due to a sparsity of information. Across the 28 remaining subjects, a total of 12 trials (0.07%) were excluded from analysis due to administrative or experimental error (e.g., noise masked subject’s response, experimenter cued subject, or audio recording cut out).

To characterize task performance at baseline between the rTMS randomization groups, we conducted a series of *t* tests. To evaluate direct and immediate changes in naming following treatment, we conducted an analysis of variance (ANOVA) to assess differences between each individual’s agent noun accuracy based on time (pre- vs. immediately post-treatment), rTMS (active vs. sham rTMS), and their interaction. This model was applied on data in a normal distribution per Q-Q test. A parallel ANOVA, motivated by transparency, assessed whether overall PNT naming accuracy differed across time, with predictors included for time, rTMS, and their interaction.

For our primary analyses, we conducted two linear regression models in the R statistical programming environment with the lme4 package (Bates et al., 2015). An initial set of models controlled for the effects of overall aphasia severity (WAB AQ), but this covariate effect was removed due to multicollinearity and correlations between overall severity and each parameter weight. Specifically, overall aphasia severity (WAB AQ) at baseline correlated with both baseline *s-weight* (*r* = 0.66, *p* < 0.001) and *p-weight* (*r* = 0.34, *p* < 0.001) and multicollinearity was determined by calculating the variance inflation factor (VIF) for WAB AQ (VIF = 46.67). Refer to the Supporting Information and Discussion for additional details. The statistical choice to not include WAB AQ was also made in line with the primary goal of this research, which was to characterize predictors of rTMS effects associated with specific stages of lexical retrieval deficits, rather than global language ability more broadly.

In addition, both parameter weights were not included in the same model due to multicollinearity between predictors. Baseline *s-weight* and *p-weight* were significantly correlated (*r* = 0.11, *p* < 0.001). Multicollinearity was determined by calculating VIFs for baseline *s-weight* (VIF = 35.29) and baseline *p-weight* (VIF = 11.31) as predictors in the same model. High multicollinearity, reflected as VIF >10, can lead to inflated standard errors, unstable coefficient estimates, and reduced model interpretability. Thus, separate models for each parameter weight were used to reduce the risk of multicollinearity-induced bias.

Parallel model structures were used for each parameter weight model. The outcome measure was proportional improvement in naming, calculated by dividing pre-to-post treatment changes by the potential improvement at baseline per subject (Fridriksson et al., 2019). rTMS stimulation condition (active vs. sham rTMS) was a between subject variable, whereas timepoint (3 and 6 months post-treatment) was a within-subject variable. Baseline parameter weight (*s-weight* and *p-weight* calculated from the PNT) was included as an interaction variable. The fixed effects structure was identical in both models, which included two interaction effects: the rTMS condition by timepoint interaction and the rTMS condition by baseline parameter weight interaction. More complex model structures failed to converge. For planned post hoc tests, we used the emmeans package and the Tukey adjustment to correct for multiple comparisons (Lenth et al., 2022).

Finally, to evaluate the performance of the linear regression models, we applied leave-one-out cross-validation. In this approach, each observation was iteratively left out of the training set, and the model was trained on the remaining *N* − 1 observations. The model was then tested on the omitted observation, and this process was repeated for all observations. This technique is well suited for small sample sizes. The resulting model performance metrics, such as root mean square error (RMSE) and adjusted *R*^2^, reflect the model’s true predictive power based on independent data at each iteration rather than inflated results from potential overfitting.

## RESULTS

Task performance for each participant is reported in Table 2, including individual *s-* and *p-weight* parameters at baseline, PNT accuracy at all timepoints, and proportional naming improvement at each follow-up visit. Four participants did not complete the 3-month follow-up timepoint. We first report basic analyses that characterize our participant groups and variables of interest. We then report the primary analysis results in the subsequent sections.

**Figure 3.**
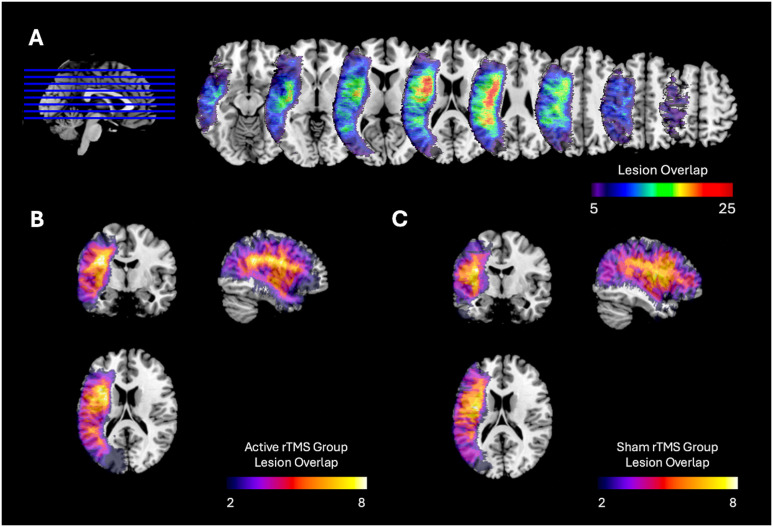
Lesion distribution maps. (A) Lesion distribution for all participants. (B) Lesion distribution for active rTMS group (*N* = 17; MNI 50, 120, 90). (C) Lesion distribution for sham rTMS group (*N* = 11; MNI 50, 120, 90). There was no significant difference between rTMS randomization groups in terms of overall lesion volume (*t* = 0.56, *p* = 0.57) or proportion of left pars triangularis lesion (i.e., the homologue of the rTMS site; *t* = 0.98, *p* = 0.33).

There were no significant differences between rTMS condition groups at baseline on overall aphasia severity (WAB AQ; *t* = 0.17, *p* = 0.87), naming ability (PNT; *t* = 1.35, *p* = 0.19), or either parameter weight (*s-weight*: *t* = 1.25, *p* = 0.22; *p-weight*: *t* = 1.55; *p* = 0.13). There was also no significant difference between rTMS groups in terms of overall lesion volume (*t* = 0.56, *p* = 0.57) or proportion of left PTr lesion (i.e., the homologue of the rTMS site; *t* = 0.98, *p* = 0.33). Figure 3 shows the lesion distributions of each rTMS group.

**Figure 4.**
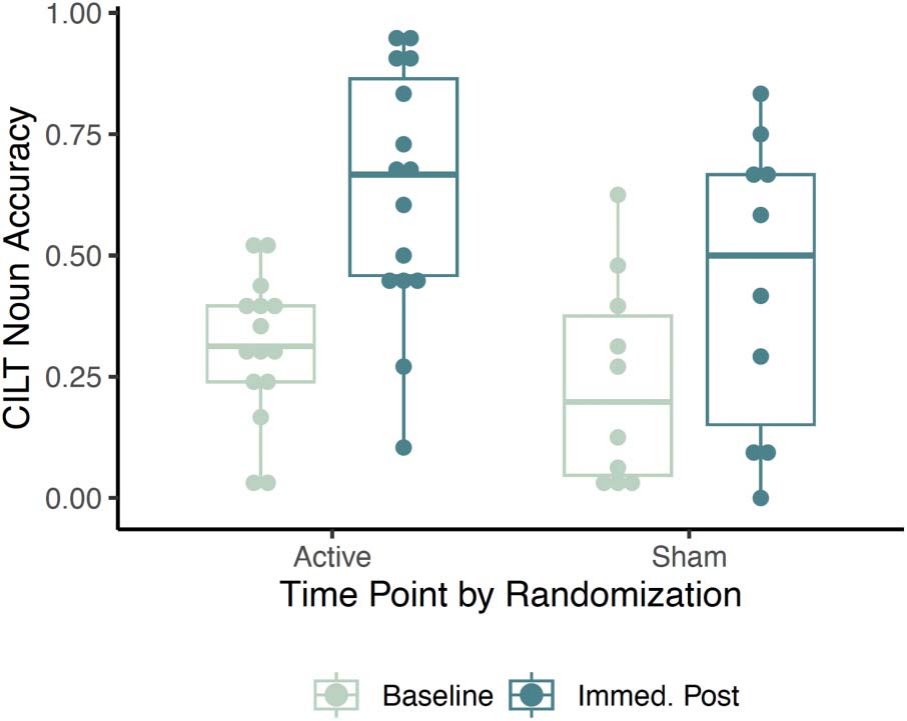
Treated items noun accuracy improvement by rTMS condition.

An ANOVA assessed immediate treatment gains for items trained during mCILT for each rTMS randomization group. Analyses confirmed a significant effect of time on agent noun accuracy (*F*(1, 22) = 17.447, *p* < 0.001), such that on average, all participants showed naming improvement after mCILT treatment, compared to pre-treatment baseline performance. Neither the main effect of rTMS (*F*(1, 22) = 3.856; *p* = 0.055) nor the rTMS × Time interaction were significant (*F*(2, 44) = 0.042; *p* = 0.384). Figure 4 shows this raw data. A separate ANOVA assessed whether PNT naming accuracy differed across time. Analyses confirmed a significant main effect of time (*F*(2, 44) = 5.258; *p* = 0.009), indicating improvement on overall PNT performance post-treatment compared to baseline. Neither the main effect of rTMS (*F*(1, 22) = 2.323; *p* = 0.142) nor the rTMS × Time interaction were significant (*F*(2, 44) = 0.626; *p* = 0.539). In spite of these general results, group level averages in PNT accuracy indicate numeric improvement from baseline to both 3- and 6-month assessments for the active rTMS randomization group, but only from baseline to 6 months post-treatment for the sham rTMS group. Proportional naming improvement, which accounts for individual baselines, were all positive, suggesting average improvement in naming. Both active rTMS and sham rTMS groups showed small proportional improvements at 3 months post-treatment (active rTMS: *M* = 0.02, *SD* = 0.27; sham rTMS: *M* = 0.02, *SD* = 0.22), whereas a greater proportional improvement was observed at 6 months for the active rTMS group (active rTMS: *M* = 0.15, *SD* = 0.29; sham rTMS: *M* = 0.06, *SD* = 0.38). Raw data are reported in Table 2. Substantial individual variability in naming outcomes supports the primary analysis approach to investigate the contributions of *s-weight* and *p-weight* parameters.

### *S-Weight* Model Results

**Figure 5.**
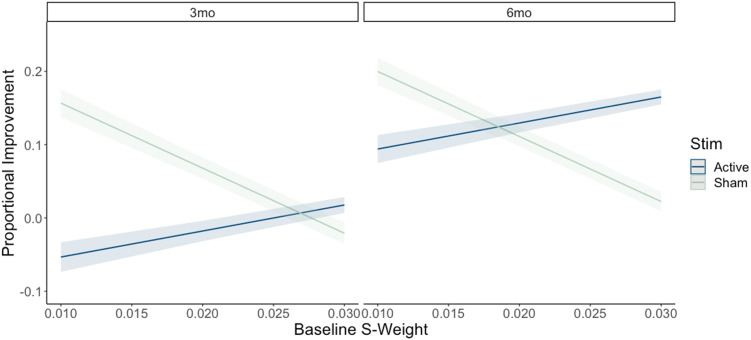
TMS by *s-weight* interaction model results. Shading indicates 95% CI. Stim = rTMS randomization condition; 3mo = 3 months post-treatment; 6mo = 6 months post-treatment.

The first model examined the effects of baseline *s-weight* on the efficacy of rTMS on long-term proportional improvement in naming (see Figure 5). Raw data for individual task performance is reported in Table 2. The full model results, including main effects and interactions, can be found in Table 4. As hypothesized, analyses confirmed a significant main effect of TMS condition (*χ*^2^(3) = 46.19, *p* < 0.001), such that on average, participants showed greater proportional naming improvement following active TMS compared to sham TMS. Moreover, we found significant interactions between rTMS condition and time (*χ*^2^(1) = 5.78, *p* < 0.001), as well as between rTMS condition and baseline *s-weight* (*χ*^2^(1) = 32.26, *p* < 0.001). Leave-one-out cross-validation results indicated moderate predictive accuracy, where some variability in the data remaining unexplained (RMSE = 0.283, *R*^2^ = 0.089, mean absolute error [MAE] = 0.220). Residual box plots revealed there were no systematic differences in how well the model fit each rTMS group due to the active to sham 2:1 randomization method implemented in the current study (see Figure S1 in the Supporting Information).

**Table 4.** *S-weight* linear regression model results

	*β*	*SE*	*t* value	*p* value	*η*^2^ (partial)
(Intercept)	0.245	0.013	18.448	<0.001	
rTMS	−0.334	0.019	−17.265	<0.001	0.008
Time	0.043	0.010	4.487	<0.001	0.030
Baseline *S-weight*	−8.879	0.452	−19.632	<0.001	0.006
rTMS : Time	0.104	0.012	8.493	<0.001	0.008
rTMS : *S-weight*	12.430	0.620	20.057	<0.001	0.040

*Note*: Time is 3 months post-treatment versus 6 months post-treatment.

*β* = estimate; *SE* = standard error; *η*^2^ (partial) = effect size measure; rTMS = active versus sham conditions.

Planned post hoc analyses revealed that rTMS interacted with time such that there were significant increases in proportional naming improvement between 3 and 6 months post-treatment for participants in the active rTMS condition (*β* = 0.147, *SE* = 0.008, *t* = 19.385, *p* < 0.001). There was a smaller but still significant increase across time for participants in the sham rTMS condition (*β* = 0.043, *SE* = 0.009, *t* = 4.487, *p* < 0.001), with the active rTMS group performing significantly better than sham at the 6-month timepoint (*β* = 0.120, *SE* = 0.008, *t* = 14.274, *p* < 0.001), but there was no difference at 3 months (*β* = 0.016, *SE* = 0.009, *t* = 1.690, *p* = 0.329).

Planned post hoc analyses also confirmed that when averaged across time, there was a significant interaction between rTMS condition and baseline *s-weight*. An analysis of simple slopes was conducted to further characterize how the effect of *s-weight* on proportional naming improvement differed between the active and sham rTMS conditions. For participants receiving active rTMS, a positive slope was observed (*β* = 3.55, *SE* = 0.424, 95% CI [2.72, 4.38]), suggesting that higher *s-weight* was associated with greater proportional improvements in naming ability. Conversely, for participants in the sham rTMS condition, a negative slope was found (*β* = −8.88, *SE* = 0.452, 95% CI [−9.77, −7.99]), indicating that higher *s-weight* was associated with lesser proportional improvements in naming ability.

### *P-Weight* Model Results

**Figure 6.**
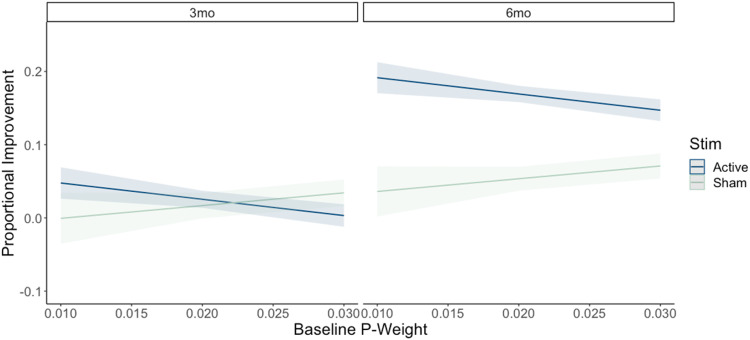
TMS by *p-weight* interaction model results. Shading indicates 95% CI. Stim = rTMS randomization condition; 3mo = 3 months post-treatment; 6mo = 6 months post-treatment.

The second model examined the effects of baseline *p-weight* on the efficacy of rTMS on long-term proportional improvement in naming (see Figure 6). Raw data for individual task performance is reported in Table 2. The full model results, including main effects and interactions, can be found in Table 5. Parallel to the *s-weight* analysis, the *p-weight* model also confirmed a main effect of TMS condition (*χ*^2^(3) = 12.15, *p* < 0.001), such that on average, participants showed greater proportional naming improvement following active TMS compared to sham TMS. Of note, we found significant interactions between rTMS condition and time (*χ*^2^(1) = 6.13, *p* < 0.001), as well as between rTMS condition and baseline *p-weight* (*χ*^2^(1) = 0.75, *p* = 0.003). Leave-one-out cross-validation results indicated limited predictive accuracy, with a substantial portion of variance in the outcome remaining unexplained (RMSE = 0.290, *R*^2^ = 0.044, MAE = 0.223). Residual box plots revealed there were no systematic differences in how well the model fit each rTMS group due to the active to sham 2:1 randomization method implemented in the current study (see Figure S2).

**Table 5.** *P-weight* linear regression model results

	*β*	*SE*	*t* value	*p* value	*η*^2^ (partial)
(Intercept)	−0.018	0.028	−0.641	0.522	
rTMS	0.088	0.033	2.649	0.008	0.007
Time	0.037	0.010	3.722	<0.001	0.030
Baseline *P-weight*	1.743	1.100	1.584	0.113	<0.001
rTMS : Time	0.107	0.013	8.537	<0.001	0.008
rTMS : *P-weight*	−3.967	1.325	−2.994	0.003	0.001

*Note*: Time is 3 months post-treatment versus 6 months post-treatment.

*β* = estimate; *SE* = standard error; *η*^2^ (partial) = effect size measure; rTMS = active versus sham conditions.

Planned post hoc analyses revealed that rTMS interacted with time such that there were significant increases in proportional naming improvement between 3 and 6 months post-treatment for participants in the active rTMS condition (*β* = 0.144, *SE* = 0.008, *t* = 18.503, *p* < 0.001). There was a smaller but still significant increase across time for participants in the sham rTMS condition (*β* = 0.037, *SE* = 0.010, *t* = 3.722, *p* = 0.001), with the active rTMS group performing significantly better than sham at the 6-month timepoint (*β* = 0.102, *SE* = 0.009, *t* = 11.834, *p* < 0.001), but there was no difference at 3 months (*β* = −0.005, *SE* = 0.009, *t* = 0.550, *p* = 0.947).

Planned post hoc analyses also revealed differing slopes for the active and sham rTMS conditions as a function of *p-weight* when averaged across time. For participants receiving active rTMS, a significant negative slope was observed (*β* = −2.22, *SE* = 0.739, 95% CI [−3.67, −0.78]), indicating that higher *p-weight* was associated with smaller proportional improvements in naming. In contrast, for participants in the sham rTMS condition, a nonsignificant positive slope was found (*β* = 1.74, *SE* = 1.100, 95% CI [−0.41, 3.90]), suggesting no robust relationship between phonological ability and naming improvements due to the confidence interval including zero.

## DISCUSSION

rTMS has shown promise in conjunction with speech-language therapy for aphasia (Li et al., 2015; Otal et al., 2015; Ren et al., 2014), yet there remains immense variability in its efficacy at the level of individual participants (Heikkinen et al., 2019; Martin et al., 2014). In this study, we tested whether individual differences in psycholinguistic stages of lexical retrieval ability may contribute to variability in long-term naming improvement, which is a significant clinical outcome given the prevalence of word retrieval deficits across all aphasia types and severities (Laine & Martin, 2006, ix, 172). In a sample of individuals with chronic post-stroke aphasia, we found on average that a 10-day course of low-frequency rTMS to rPTr in conjunction with mCILT improved long-term naming more than mCILT alone. Furthermore, both individual baseline semantic and phonological abilities informed the efficacy of rTMS on proportional improvement naming outcomes at 3 and 6 months post-treatment. We discuss the details and implications of these findings below.

First, as shown in prior work, mCILT was efficacious, as demonstrated by the finding that even individuals in the sham rTMS group showed robust naming improvements immediately following 10 days of treatment. Positive effects of mCILT on agent noun naming accuracy for treated items show the language therapy worked as intended and appropriately engaged cognitive systems related to naming, which is critical to make use of the state-dependent effects of rTMS (Cherney et al., 2008; Harvey & Hamilton, 2022). Immediate results thus showed that mCILT was efficacious. Because the mCILT items were strategically chosen to avoid overlap with the target words on the PNT, we were able to evaluate not just maintenance of naming improvements, but also generalization to a distinct, untrained set of nouns. However, a simple analysis of PNT change revealed that although overall naming accuracy differed across time, there was not a main effect of rTMS condition nor an interaction between rTMS and time. This indicates overall improvement of PNT performance following mCILT compared to baseline, regardless of rTMS. The lack of immediate rTMS effects on naming may be attributed, at least in part, to the strong effects of mCILT eclipsing relatively small rTMS effects; but this remains a topic for further investigation. Although our study did not replicate prior findings of immediate rTMS effects on naming, this leads to our first research question: whether rTMS can help sustain or enhance the effects of mCILT for naming improvement maintained at long-term timepoints.

Indeed, we found evidence supporting the hypothesis that low-frequency rTMS to rPTr in conjunction with mCILT improves long-term proportional naming improvement more than mCILT alone. Critically, despite the absence of immediate effects of rTMS on treatment outcomes, long-term maintenance effects were observed. This was shown by robust main effects of rTMS condition in both models: while holding timepoint and baseline parameter weights at average for this sample, participants demonstrated significantly greater proportional naming improvement in the active compared to sham rTMS condition. Furthermore, these effects increased from 3 to 6 months post-treatment, consistent with accounts that the effects of rTMS are reinforced over time (Hamilton et al., 2010; Harvey et al., 2017; Medina et al., 2012). These findings are also consistent with prior research demonstrating that rTMS can enhance naming abilities in chronic stages of post-stroke aphasia (Hong et al., 2021; Li et al., 2015; Otal et al., 2015; Ren et al., 2014). It is important to contrast the current findings with the lack of overall rTMS effects in the ANOVA analyses, which did not account for the psycholinguistic locus of deficit, or the “room for growth” captured by proportional improvement computations. It is possible that rTMS effects are best observed at maintenance timepoints, rather than immediately post-treatment. Although prior work has posited the potential for long-term circuit strengthening with rTMS (Barwood et al., 2013; Hamilton et al., 2010; Harvey et al., 2017; Nudo, 2011), our data indicate that any such benefits may be gradual and not necessarily observable immediately post-treatment. Furthermore, our findings of naming improvement may also be best observed when assessed with proportion of maximum gain. There are both advantages (e.g., no inflation from high baseline performance) and disadvantages (e.g., a ceiling effect on potential improvement) in the use of improvement as a proportion of maximum gain compared to absolute improvement. In support of data transparency, we include Supporting Information for identical analyses on absolute naming outcomes. Future studies are needed to replicate the current findings and to additionally examine multivariate sources of rTMS response variability, including size and specific location of neural damage (Martin et al., 2009), genetic and neurophysiological factors of neuroplasticity (Dresang et al., 2022), and psycholinguistic loci of lexical impairments as suggested here.

Furthermore, we found preliminary evidence that individual baseline semantic and phonological abilities influence the efficacy of rTMS on long-term naming outcomes. Main effects revealed greater baseline semantic and phonological strength were associated with greater proportional improvement in naming ability at both post-treatment timepoints. This effect was generally larger for participants who received active rTMS to rPTr coupled with mCILT when compared to sham rTMS. Importantly, significant interaction effects demonstrate that the baseline loci of naming deficits influenced the efficacy of rTMS on long-term naming. In particular, greater *s-weight* was associated with greater proportional naming improvement following active rTMS paired with mCILT, and with reduced likelihood of proportional improvement following mCILT alone (sham rTMS condition). These trends were consistent across 3- and 6-month timepoints post-treatment. In addition, the *s-weight* analysis found a significant interaction between rTMS condition and time, such that proportional improvement was greater at 6 months compared to 3 months for the active rTMS group, while those who received sham showed no significant difference. On the other hand, when averaged across time, the interaction between *p-weight* and rTMS condition suggested that phonological ability may moderate the impact of active rTMS on naming outcomes, with greater *p-weight* associated with diminished benefits from active rTMS. The *p-weight* analysis also revealed a robust interaction between rTMS and time post-treatment. Specifically, at 6 months post-treatment, individuals who received active rTMS prior to mCILT showed significantly greater proportional naming improvement than those who received sham rTMS. Although the effect sizes observed in both models are small, they are nonetheless significant. In addition, the cross-validation results indicate that our models avoided overfitting the data, but there remains a substantial amount of variance in naming outcomes that is still unexplained. Future investigations should continue to pursue larger sample sizes for robust power to detect the effects investigated here and account for other factors that explain additional variance.

Taken together, these results indicate that active rTMS specifically supports sustained improvements in long-term naming ability, even in the absence of immediate rTMS effects, and these long-term effects are critically associated with baseline semantic and phonological abilities. In particular, these findings highlight the importance of semantic stages of lexical retrieval and potential cognitive reserves associated with maximal benefits from rTMS on long-term naming outcomes. These results are broadly consistent with findings that lexical access stages of word retrieval may be differentially influenced by rTMS of rPTr (Harvey et al., 2019; Medina et al., 2012). As one example, Harvey and colleagues (2019) found continuous theta burst stimulation (cTBS) of rPTr was associated with greater naming improvements as a function of the degree of phonologic naming impairment (defined as proportion of any kind of phonological error) at baseline, while this was not true for semantic errors. As a second example, Medina and colleagues (2012) found cTBS of rPTr was associated with significant increases in lexical-semantic aspects of discourse production, such as number of unique nouns and total verbs produced. These two examples are partially consistent with the current results, suggesting lexical access stages of word retrieval may be differentially affected by rTMS. However, there are also important differences between studies. For instance, due to a higher prevalence of phonological errors relative to semantic errors in Harvey et al.’s sample, there may be different semantic results compared to the current findings because of insufficient power. Medina and colleagues assessed discourse production and did not have direct measures of semantic versus phonological error types. In addition, the goals of our research were to examine the predictive power of baseline lexical access abilities, rather than to directly determine which stages improved following rTMS of rPTr. Like many prior studies, Harvey et al. (2019) and Medina et al. (2012) were limited by smaller sample sizes, an absence of language treatment, and no long-term outcome measures. These factors, along with different operationalization of error types and lexical-semantic ability, may contribute to differences in semantic versus phonological findings between studies.

Furthermore, the current findings provide evidence in support of mechanistic accounts by which low-frequency stimulation to rPTr supports long-term language improvement. In particular, these results support the hypothesis that the strength of semantic access is positively associated with the efficacy of rTMS to rPTr on naming outcomes. This is largely in line with findings from Medina et al. (2012) related to semantically driven discourse production improvements. It is also consistent with evidence that rTMS of rPTr in chronic aphasia induces N400 signal changes (Barwood et al., 2011), which are commonly associated with lexical-semantic aspects of language processing (Kutas & Federmeier, 2000; Lau et al., 2008). Taken together, these results support the account that low-frequency rTMS of rPTr may enhance lexical-semantic activation during word retrieval or that semantic abilities may confer better rTMS response, as indicated here. While our findings help to characterize the aspects of word retrieval that contribute to variability in sustained rTMS naming outcomes, our results do not resolve ongoing debate regarding the specific neural mechanisms by which rTMS of rPTr facilitates language improvement. In addition to the hypotheses presented in the Introduction, it is also possible that rPTr may be implicated in a domain-general network that contributes to language abilities via cognitive control mechanisms. Our findings could be in line with the hypothesis that rPTr plays a role in resolving competition between conflicting linguistic representations (Novick et al., 2010). It is plausible that rPTr is part of a domain-general compensatory mechanism that relies on cognitive processes such as inhibition and attention control to facilitate successful naming (Turkeltaub et al., 2011). However, the current study design is unable to speak directly to this hypothesis.

Regardless of the specific mechanism, this study critically contributes to a growing body of evidence that naming improvements can increase over time following rTMS, even in the absence of ongoing therapy (Barwood et al., 2013; Hamilton et al., 2010; Harvey et al., 2017; Naeser et al., 2005). As one example, Barwood and colleagues (2013) examined long-term language recovery subsequent to low-frequency rTMS to homologous language sites to modulate language in chronic non-fluent aphasia. In their study of 12 participants (six active rTMS and six sham rTMS), they observed treatment-related changes up to 12 months post-stimulation in the active stimulation group compared to the placebo control group. Their study findings extended to naming performance, expressive language, and auditory comprehension. Although this study’s procedures were similar in that they involved 20 minutes of rTMS for 10 days, with the target being the rPTr (Broadmann area 45), no speech-language therapy was conducted. A more recent study by Georgiou and Kambanaros (2022) compared the long-term efficacy of low-frequency rTMS versus cTBS as standalone chronic aphasia treatments. Both procedures similarly targeted rPTr. They found both stimulation paradigms resulted in improvement trends across several language abilities, including naming, reading, verbal receptive language, and expressive language. Furthermore, one of their six total participants received follow-up testing 2 years post-treatment. This subject, who received cTBS, maintained the treatment improvements that were documented immediately after the stimulation treatment. Taken together, there is growing evidence that inhibitory rTMS over the rPTr, especially when coupled with efficacious speech-language therapies like mCILT, has the potential to drive neuroplastic changes that support language recovery in chronic post-stroke aphasia.

It will be vital for future studies to test the generalizability of the current findings to other language treatments for aphasia. Although mCILT was selected to avoid treatments that uniquely target semantics or phonology, the stimuli did capitalize on semantic associations between agents, verbs, and patients (e.g., *jester–juggling–balls*). It is possible that *s-weight* would be a strong predictor of mCILT or other interventions centered on semantic activation, such as semantic feature analysis (Boyle & Coelho, 1995) or verb networks strengthening treatment (Edmonds, 2016). In contrast, *p-weight* may be a better predictor of outcomes following interventions focused on phonology, such as phonomotor treatment (Kendall et al., 2015). Details of the language treatment protocols are likely to influence the strength of baseline ability predictors, such as a main effect of *s-weight* but not *p-weight* in the current analysis potentially because strong semantic abilities supported participants’ likelihood of improving from a treatment that used semantically associated words.

It is important to note that both *s-weight* and *p-weight* scores at baseline were correlated with overall aphasia severity, as measured by WAB AQ. Controlling for overall severity in the analyses resulted in the same robust interactions reported above (see Supporting Information), indicating that semantic and phonological abilities are robust predictors even when controlling for the extent of overall language impairment. However, to reduce multicollinearity concerns, we report models that did not include aphasia severity and examine independent effects of *s-weight* and *p-weight* in separate models. This is supported by prior evidence of correlation, and potential interdependence, of naming error types. An investigation of naming error patterns from a large aphasia database (Moss Aphasia Psycholinguistics Project Database, *N* = 296; Mirman et al., 2010) indicates that there is a moderate positive correlation between semantic and phonological abilities when controlling for aphasia severity, and that both semantic and phonological ability influence overall naming (Jebahi et al., 2024). Correlations between *s-weight*, *p-weight*, and aphasia severity may reflect the fact that semantic and phonological abilities are both important components of overall language functions that are commonly affected by aphasia.

Compared to measures of gross accuracy, error type parameters provide more detailed information about the mechanisms of word retrieval improvement. However, error type metrics like *s-weight* and *p-weight* are potentially interdependent since they are derived from a finite set of data that shares variance. It is plausible that deficits in one stage of lexical retrieval would influence adjustments or compensations in the other (e.g., Dresang et al., 2024). For example, a participant with few semantic errors might show increased relative influence or visibility of phonologic errors, or vice versa. A limitation of the current results is that they cannot speak to how semantic and phonological errors may interact. Another limitation of error type measures is that the errors could result from breakdowns at multiple component processes. For example, semantic errors could be attributed to compromised integrity of semantic representations or reduced strength of semantic access and retrieval. Similarly, phonological errors may reflect reduced strength of phonological access and retrieval or deficits in motor speech and phonetic production ability. Thus, a possible alternative explanation for the current findings is that rTMS facilitated naming improvements not by enhancing semantic or phonological access, but rather by remediating motor-speech deficits or directly strengthening semantic representations. Future investigations may provide greater insight by examining multiple semantic and phonological assessments as well as investigating shared variance between error types in a joint analysis with a greater number of observations (e.g., Jebahi et al., 2024).

Future work should investigate independent and comprehensive measures of semantic versus phonological abilities, as well as potential trade-offs that may occur between semantic and phonological processes and cognitive control. Larger sample sizes or greater number of trials would be necessary to examine complex interactions between semantic and phonological error types. With greater statistical power, more complex model structures may also integrate multiple post-treatment timepoints, control for factors like overall aphasia severity, and examine three-way interactions between each parameter weight and rTMS conditions. Nonetheless, for a study that requires an intensive duration of sentence-level language therapy combined with rTMS, we have one of the largest aphasia sample sizes to date. Another limitation of the current dataset is that the participant sample did not represent individuals with very severe or very mild (latent) aphasia, so the reported results may not generalize to individuals with relative extreme presentation across the severity spectrum. However, the reported sample is highly illustrative of the group of individuals with aphasia that would most likely be recommended for mCILT as a speech-language treatment. This further justifies our exclusion of two participants with relatively severe aphasia who did not produce sufficient meaningful errors to characterize their loci of lexical retrieval deficits. Finally, interpretation of long-term effects must be tempered by the possibility that outcomes may be influenced by confounding factors between the end of treatment and the follow-up assessments. Although no participants received speech-language services during this time, individual environmental and social factors may have varied.

These findings may also contribute to identification of critical mechanisms that can be leveraged for aphasia treatment. One hypothesis is that rTMS enhances use-dependent neuroplasticity in aphasia rehabilitation, which supports ongoing language improvements as individuals continue to use language in their daily lives. Such ongoing, use-dependent mechanisms may underpin the sustained long-term outcomes observed here. It is possible that these functional changes are supported by rTMS-induced transcallosal disinhibition as well as changes in the bilateral PTr network (e.g., Harvey et al., 2017). There is evidence supporting a shift in the role of rPTr post-stroke, indicating that rPTr may become a critical region for integration and segregation of communication across neural networks, which in turn has been associated with reduced phonological naming errors in individuals with chronic aphasia (Stoll et al., 2023). However, further work must investigate these mechanisms more closely by implementing such procedures to test predictions of dual stream neural networks associated with semantic and phonological stages of lexical retrieval (Hula et al., 2020; Saur et al., 2008).

### Conclusion

Low-frequency rTMS to rPTr in conjunction with mCILT improved naming more than mCILT alone between 3 and 6 months post-treatment. Furthermore, individual differences in baseline semantic and phonological stages of lexical retrieval were substantial predictors of rTMS efficacy on long-term naming outcomes; this is the case where main effects showed that greater baseline semantic and phonological strength were both associated with greater proportional improvement in naming ability, and to a larger extent in participants who received active rTMS compared to sham rTMS. Individuals with greater *s-weight* showed greater naming improvement following active rTMS than sham, and this proportional improvement was greater at 6 months compared to 3 months for the active rTMS group, while the sham group showed no difference across time. In contrast, individuals with greater *p-weight* who received active rTMS compared to sham showed significantly greater naming improvement specifically at 6 months post-treatment. This study is among the first in a larger sample to demonstrate that baseline linguistic characteristics of individual participants with aphasia contribute to variability in sustained rTMS and aphasia treatment outcomes. These findings may have significant implications for clinical decision making as well as mechanistic accounts of noninvasive brain stimulation adjuvants to neurorehabilitation.

## ACKNOWLEDGMENTS

We wish to express our gratitude to Adelyn Brecher for providing support in data coding.

## FUNDING INFORMATION

H. Branch Coslett, National Institute on Deafness and Other Communication Disorders (https://dx.doi.org/10.13039/100000055), Award ID: R01DC016800. Eunice Kennedy Shriver National Institute of Child Health and Human Development (https://dx.doi.org/10.13039/100009633), Award ID: T32HD071844.

## AUTHOR CONTRIBUTIONS

**Haley C. Dresang**: Data curation, Formal analysis, Investigation, Methodology, Visualization, Writing – original draft. **Denise Y. Harvey**: Data curation, Investigation, Methodology, Project administration, Writing – original draft. **Leslie Vnenchak**: Data curation, Investigation, Project administration. **Shreya Parchure**: Formal analysis, Visualization. **Sam Cason**: Investigation. **Peter Twigg**: Investigation. **Olu Faseyitan**: Investigation, Project administration. **Lynn M. Maher**: Conceptualization, Formal analysis, Methodology, Writing – review & editing. **Roy H. Hamilton**: Conceptualization, Funding acquisition, Methodology, Writing – review & editing. **H. Branch Coslett**: Conceptualization, Funding acquisition, Methodology, Supervision, Writing – review & editing.

## CODE AND DATA AVAILABILITY STATEMENT

Public access code and data are available at https://osf.io/7ahxu/. Further details may be requested from the authors.

## Supplementary Material



## TECHNICAL TERMS

Aphasia: A language disorder caused by brain damage, such as stroke, which can affect language production, comprehension, reading, and writing.
Transcranial Magnetic Stimulation (TMS): A noninvasive brain stimulation technique that uses magnetic fields to modulate brain activity for research or therapeutic purposes.
Semantics: The study of meaning in language, including how words, phrases, and sentences represent ideas and concepts.
Phonology: The study of the sound system of a language, focusing on the patterns and rules governing speech sounds.
Constraint-Induced Language Therapy (CILT): A rehabilitation approach that encourages the use of spoken language by restricting alternative communication methods.
