# Supplementary material for: Semantic and Phonological Abilities Inform Efficacy of Transcranial Magnetic Stimulation on Sustained Aphasia Treatment Outcomes
Source: Neurobiol Lang (Camb). 2025 Mar 7;6:nol_a_00160. doi: 10.1162/nol_a_00160 (PMC11932577; doi:10.1162/nol_a_00160)

SUPPLEMENTAL MATERIALS

Table S1. Checklist for mCILT treatment fidelity.

Checklist for Treatment Fidelity: rTMS + mCILT in aphasia treatment sessions					
	Clinician reviewed:	Scoring: 4 = always 3 = mostly; 2 = sometimes; 1 = rarely; 0 = never			
	Reviewer:				
	Checklist Items	Session #		Session #	
		Observations	Score	Observations	Score
1	Probe administered correctly (when applicable) without feedback on accuracy or results				
2	Barrier placed appropriately				
3	Review of target items at the start of a new set/level				
4	Self-cuing materials (paper/boogie board and pen/stylus) are placed within the patient's reach (if needed)				
5	Patient uses appropriate response as per level of difficulty for each exchange				
6	Cueing hierarchy used appropriately (individualized given attempt)				
7	Patient repeats target sentence for requested items s/he does NOT have				
8	Patient repeats target sentence for requested items s/he has				
9	Patient afforded choice in session as appropriate/pt included in deciding how to proceed (e.g., which sets to practice, when to receive a cue, etc.)				
10	Score sheet utilized to track progress during session				
11	Increased level appropriately based on accuracy and/or following 10-item probes every 3rd day				
12	Session ends with positive experience				
13	Difficultly level at start and end of session noted in Difficulty Tracking Sheet (placed in binder)				

		Total Score		Total Score	
	Additional Comments				

Table S2. *S-weight* linear regression model results, controlling for aphasia severity.

	β	SE	t value	p value	η² (partial)
(Intercept)	0.094	0.016	5.732	< 0.001	
rTMS	0.112	0.016	7.143	< 0.001	0.008
Time	0.397	0.020	-20.324	< 0.001	0.030
Baseline S-weight	0.033	0.010	3.452	< 0.001	0.007
Baseline WAB AQ	-13.570	0.537	-25.244	< 0.001	0.020
rTMS : Time	0.004	0.000	15.658	< 0.001	0.009
rTMS : S-weight	0.116	0.012	9.526	< 0.001	0.050

Note: β = estimate. *SE* = standard error. η² (partial) = effect size measure. rTMS = active versus sham conditions. Time = 3-months post-treatment versus 6-months post-treatment. WAB AQ = Western Aphasia Battery–Aphasia Quotient (Kertesz, 2006), measure of overall aphasia severity.

Table S3. *P-weight* linear regression model results, controlling for aphasia severity.

	β	SE	t value	p value	η² (partial)
(Intercept)	-0.088	0.030	-2.914	0.004	
rTMS	0.100	0.033	3.007	0.003	0.007
Time	0.032	0.010	3.225	0.001	0.030
Baseline P-weight	0.919	1.105	0.831	0.406	< 0.001
Baseline WAB AQ	0.001	0.000	6.417	< 0.001	0.003
rTMS : Time	0.114	0.013	9.052	< 0.001	0.009
rTMS : P-weight	-5.243	1.337	-3.921	< 0.001	0.002

Note: β = estimate. *SE* = standard error. η² (partial) = effect size measure. rTMS = active versus sham conditions. Time = 3-months post-treatment versus 6-months post-treatment. WAB AQ = Western Aphasia Battery–Aphasia Quotient (Kertesz, 2006), measure of overall aphasia severity.

Table S4. *S-weight* linear regression model results, absolute naming outcomes.

	β	SE	t value	p value
(Intercept)	0.127	0.014	8.781	< 0.001
rTMS	0.029	0.021	-1.394	0.163
Time (baseline vs 3mo)	-0.011	0.008	-1.319	0.187
Time (baseline vs 6mo)	0.030	0.008	3.873	< 0.001
Baseline S-weight	19.726	0.525	37.556	< 0.001
rTMS : Time (baseline vs 3mo)	-0.002	0.010	-0.181	0.857
rTMS : Time (baseline vs 6mo)	0.012	0.010	-1.212	0.226
rTMS : S-weight	1.904	0.721	2.640	0.008

Note: Identical model structures examined absolute naming outcomes, rather than proportional naming improvement. Results differ from proportional improvement model for main effect of rTMS, rTMS:Time interactions. β = estimate. *SE* = standard error. rTMS = active versus sham conditions. Time = 3-months post-treatment versus 6-months post-treatment.

Table S5. *P-weight* linear regression model results, absolute naming outcomes.

	β	SE	t value	p value
(Intercept)	1.836	0.034	54.242	< 0.001
rTMS	0.735	0.040	-18.365	< 0.001
Time (baseline vs 3mo)	-0.021	0.009	-2.351	0.019
Time (baseline vs 6mo)	0.035	0.008	4.195	< 0.001
Baseline P-weight	-8.508	1.348	-6.314	< 0.001
rTMS : Time (baseline vs 3mo)	-0.025	0.011	2.207	0.027
rTMS : Time (baseline vs 6mo)	0.025	0.011	-2.356	0.019
rTMS : P-weight	36.734	1.629	22.552	< 0.001

Note: Identical model structures examined absolute naming outcomes, rather than proportional naming improvement. Results differ from proportional improvement model for the intercept and main effect of Baseline *P-weight*. β = estimate. *SE* = standard error. rTMS = active versus sham conditions. Time = 3-months post-treatment versus 6-months post-treatment.

Figure S1. *S-weight* model residual box plots by rTMS group.

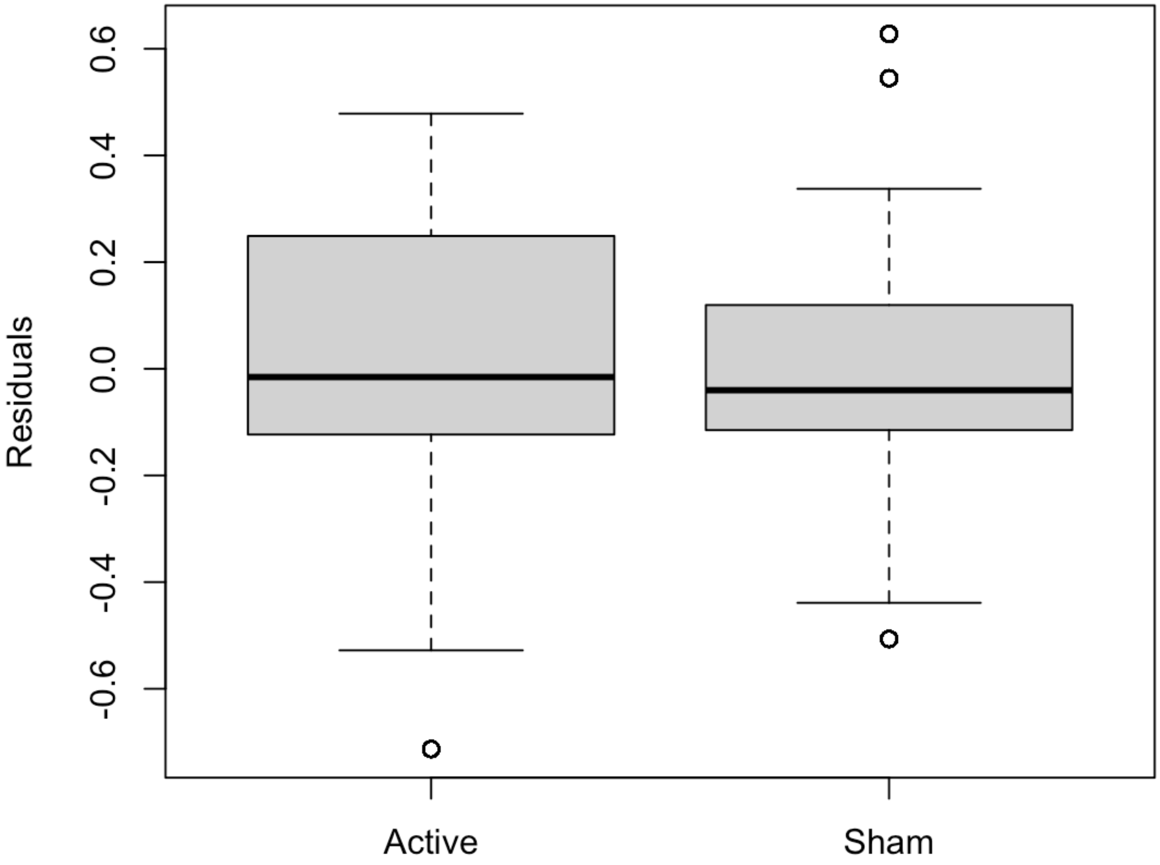

Note: There was no difference in how well the *S-weight* model fit data for each rTMS group, despite the 2:1 ratio of active:sham conditions (t < 0.001, p-value = 1.00, 95% CI = [-0.012, 0.012]).

Figure S2. *P-weight* model residual box plots by rTMS group.

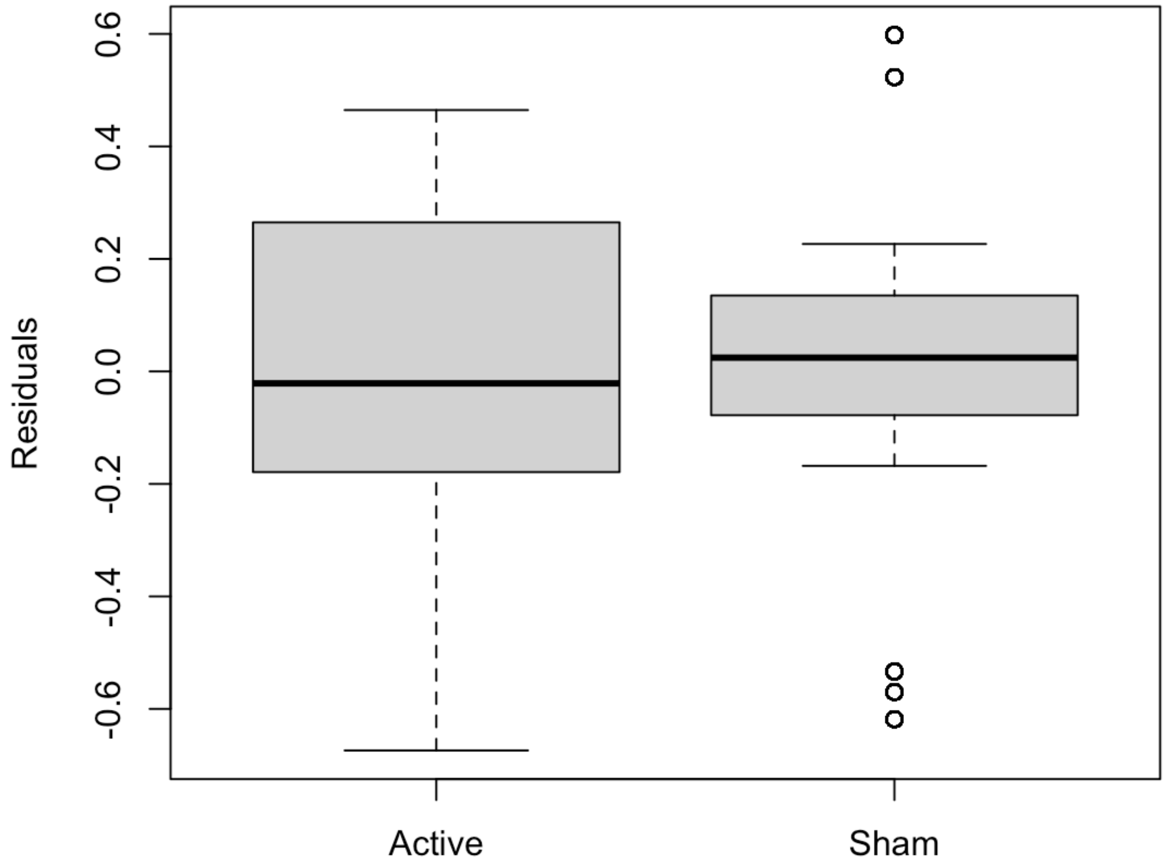

Note: There was no difference in how well the *P-weight* model fit data for each rTMS group, despite the 2:1 ratio of active:sham conditions ($t < 0.001$, p-value = 1.00, 95% CI = [-0.012, 0.012]).

Figure S3. Aphasia severity (WAB AQ) by baseline *s-weight* raw data.

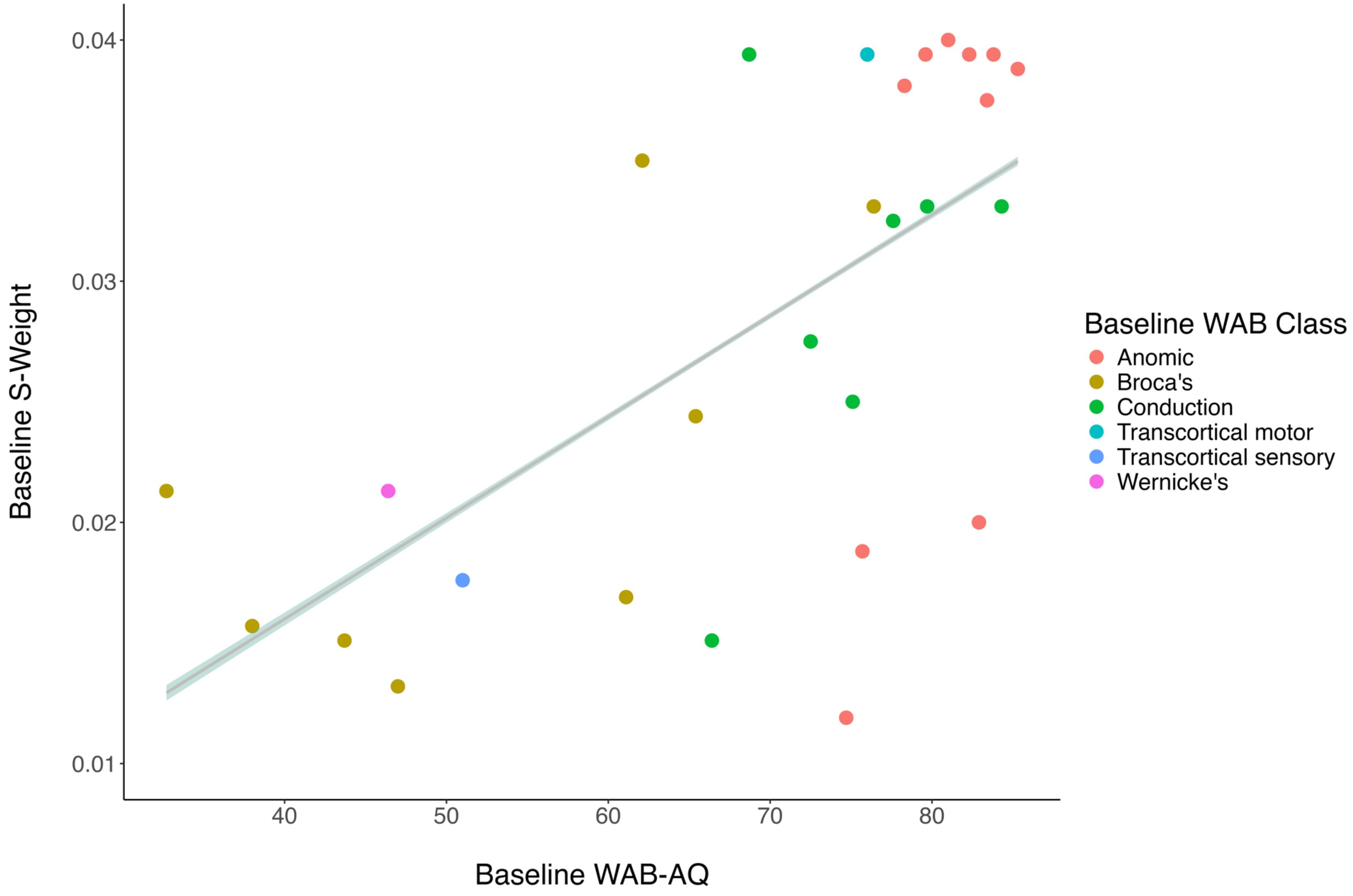

Figure S4. Aphasia severity (WAB AQ) by baseline *p-weight* raw data.

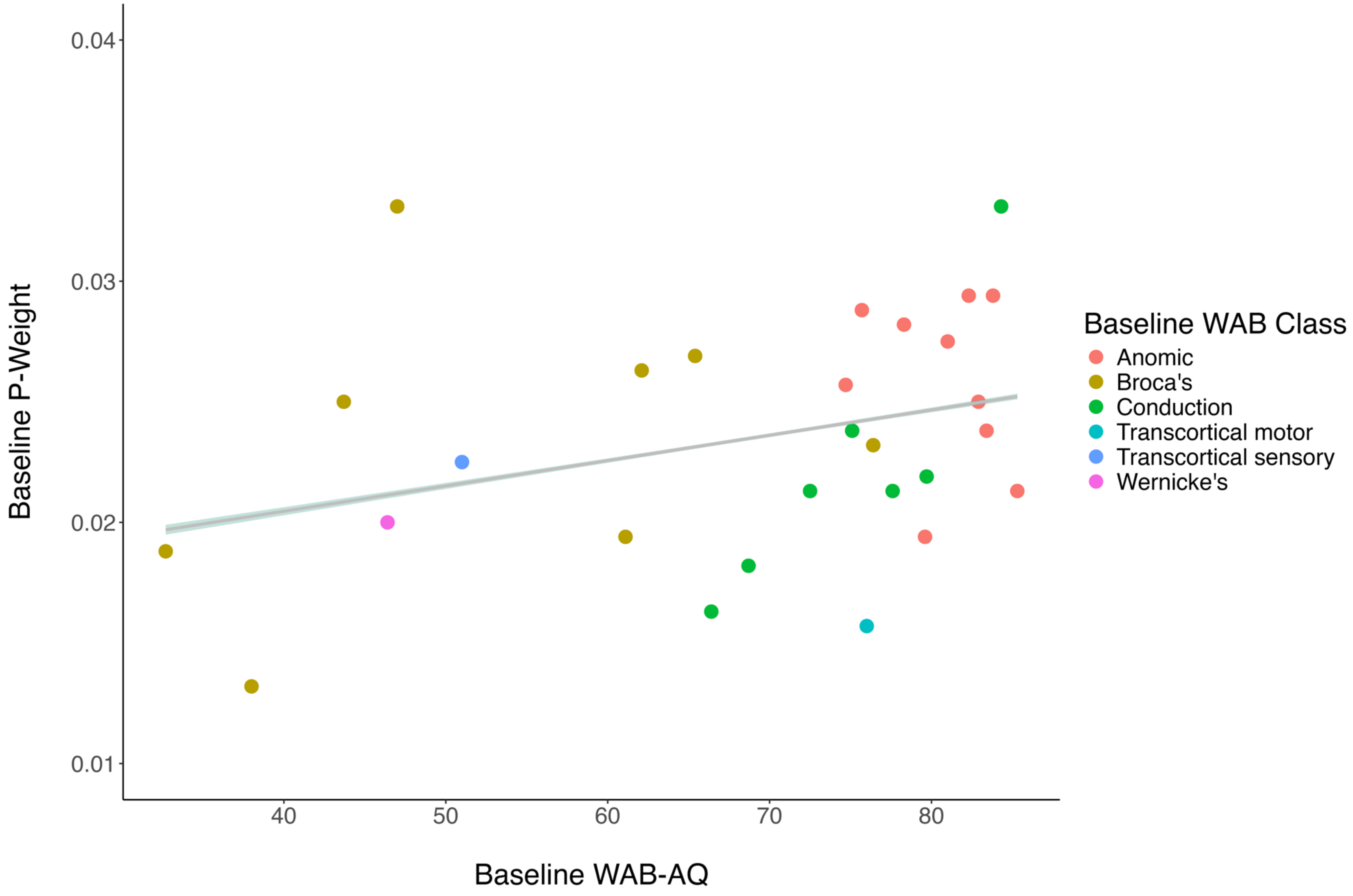

Supplement: Supplementary file 1 [file nol-6-nol_a_00160-s001.pdf]
